# Reduced Graphene Oxide Reinforces Boron Carbide with High-Pressure and High-Temperature Sintering

**DOI:** 10.3390/ma17235838

**Published:** 2024-11-28

**Authors:** Xiaonan Wang, Dianzhen Wang, Kaixuan Rong, Qiang Tao, Pinwen Zhu

**Affiliations:** 1 Key Laboratory of Functional Materials Physics and Chemistry of the Ministry of Education, Jilin Normal University, Changchun 130103, China; cjywxn@126.com; 2 Synergetic Extreme Condition High-Pressure Science Center, State Key Laboratory of Superhard Materials, College of Physics, Jilin University, Qianjin Street, Changchun 130012, China

**Keywords:** boron carbide, toughening mechanism, high pressure, high temperature

## Abstract

Introducing a second phase has been an effective way to solve the brittleness of boron carbide (B_4_C) for its application. Though reduced graphene oxide (rGO) is an ideal candidate for reinforcing the B_4_C duo’s two-dimensional structure and excellent mechanical properties, the toughness is less than 6 MPa·m^1/2^, or the hardness is lower than 30 GPa in B_4_C–graphene composites. A barrier to enhancing toughness is the weak interface strength between rGO and B_4_C, which limits the bridging and pull-out toughening effects of rGO. In this work, internal stress was introduced using a high-pressure and high-temperature (HPHT) method with B_4_C–rGO composites. The optimal hardness and toughness values for the B_4_C-2 *vol%* rGO composite reached 30.1 GPa and 8.6 MPa·m^1/2^, respectively. The improvement in toughness was 4 times higher than that of pure B_4_C. The internal stress in the composite increased gradually from 2.3 GPa to 3.3 GPa with an increase in rGO content from 1 *vol%* to 3 *vol%*. Crack deflection, bridging, and rGO pull-out are responsible for the improvement in toughness. Moreover, the high internal stress contributed to the formation of good interface strength by embedding rGO into the B_4_C matrix particles, which further enhanced the dissipation of the crack energy during the pull-out process and led to high toughness. This work provides new insights into synthesizing high-toughness B_4_C matrix composites.

## 1. Introduction

Boron carbide (B_4_C) is one of the most important structural ceramics with outstanding properties, such as high hardness values (>30 GPa), low density values (~2.52 g/cm^3^), a high Young’s modulus (~440 GPa), a high melting point, and a low wear coefficient [1,2]. Due to these properties, B_4_C ceramics have become the best candidate material for a wide range of engineering applications. However, the intrinsically strong covalent-bonding and ultra-low self-diffusion coefficient of B_4_C result in great difficulty in preparing dense B_4_C composites, leading to its Achilles’ heel of low fracture toughness and poor sinterability [3,4,5]. Therefore, mitigating these drawbacks of B_4_C is a critical issue that has an important impact on the development and application of B_4_C matrix composites.

The introduction of second-phase particles with high hardness and toughness values has been regarded as an efficient way to enhance the mechanical properties of B_4_C ceramic composites. It is well known that graphene possesses excellent properties, such as an ultra-high Young’s modulus (0.5–1 Ta), high tensile strength (130 GPa), and a large specific surface area, showcasing its broad application prospects in enhancing brittle ceramic materials [6,7]. Recently, multi-layer graphene (MLG), in the form of graphene oxide (GO) and graphene platelets (GPLs), has been used to enhance the mechanical properties of B_4_C ceramic composites [8,9,10,11,12]. Sedlák et al. reported an improved fracture toughness of 4.6 MPa·m^1/2^ for B_4_C-6 *wt%* GPL composites, but the hardness was only 19.6 GPa [8]. Tan et al. indicated that the optimal hardness and toughness values of B_4_C-4 *vol%* GNP composites reached 33.3 GPa and 5.26 MPa·m^1/2^, respectively [10]. Similarly, Li et al. used reduced graphene oxide (rGO) to reinforce B_4_C through spark plasma sintering and found that the optimal hardness and toughness values were 33 GPa and 4.88 MPa·m^1/2^, respectively, by adding 2 *vol%* of rGO [11]. Wang et al. prepared B_4_C–rGO composites under a 30 MPa pressure at 1950 °C. In their study, a sample with a 2 *wt%* rGO content achieved optimal hardness and toughness values of 33 GPa and 5.85 MPa·m^1/2^, respectively [12]. Up to now, improvements in toughness for graphene-reinforced B_4_C ceramic composites have only reached 6 MPa·m^1/2^ when maintaining a high hardness value of 30 GPa. So, determining how to take full advantage of graphene in reinforcing ceramics to acquire excellent mechanical properties is still a challenge.

Generally, an improvement in toughness for stiff ceramics can be ascribed to crack deflection, crack bridging, and the pull-out mechanism induced by a second phase [13]. In practice, the crack bridging and pull-out mechanisms of graphene depend on the interfacial strength and interface friction, which are different from other second phases [14,15]. Firstly, the friction dissipation energy of graphene has great influence on the pull-out toughening mechanism due to its large specific surface area. If the interface friction can be enhanced through the construction of internal stress in the composite during the pull-out process, then the dissipation of friction energy can be maximized. Secondly, graphene bridging exhibits distinctive characteristics of interlayer sliding and an asynchronous delamination fracture mode, which enhances energy dissipation and provides durable bridging effects [16]. Thirdly, the formation of good interfacial strength between graphene and B_4_C is difficult due to differences in their surface tension and density. Therefore, optimizing the crack bridging and pull-out of graphene may be realized by introducing internal stress.

However, the introduction of internal stress requires a suitable sintering process. For common sintering methods like hot pressing, spark plasma sintering, and hot isostatic pressing sintering are not able to provide enough driving force at the MPa level [17,18,19]. Recently, high-pressure and high-temperature (HPHT) sintering has been used to synthesize ceramic matrix composites and achieved good densification and has excellent mechanical properties [20,21,22]. Additionally, the interlayer spacing of multi-layer GO can decrease from 7–9 Å to 3–4 Å under high pressure (GPa) and heat treatment, which shows that GO may store more external stress [23]. Thus, the frictional shear stress may be higher, and a better condition of the interface between GO and B_4_C may be achieved. Up to now, the construction of internal stress in B_4_C/rGO composites using the HPHT method has not been reported.

In this work, B_4_C–rGO composites having internal stress were synthesized by the HPHT method. The influence of the rGO contents on the densification, hardness, and toughness of the B_4_C–rGO composites were systematically evaluated. Moreover, the combinations and the internal stress between rGO and B_4_C were analyzed. This work is promising for producing more robust ceramics by introducing internal stress.

## 2. Experimental Details

Commercially available B_4_C powders (particle size, 2–3 µm; purity, 99.9%; Shanghai Aladdin Co., Ltd., Shanghai, China) and GO (length, 0.5–3 µm; 1–10 layers; purity, 99.9%; Shanghai Aladdin Co., Ltd., Shanghai, China) were used as raw materials. As the reinforcing phase, GO powders with different contents (1 *vol%*, 2 *vol%*, and 3 *vol%*) were mixed with B_4_C by alcohol with an agate mortar for 3 h. SEM images of initial mixed powders with different GO content were shown in Figure S1. Then, the mixtures were heat-treated for 2 h at 80 °C in a vacuum drying oven. Thereafter, the mixed powders were cold-pressed to produce cylindrical samples with a diameter of 4 mm and a height of 2.5 mm and were sealed into an h-BN capsule. Finally, the samples were synthesized using a 6 × 14,400 KN cubic anvil HPHT apparatus. WC, with a square anvil face of 23.5 mm × 23.5 mm, was used as an anvil. A graphite tube as the heater was selected, and the temperature was calibrated using W5%Re–W26%Re thermocouples. The pressure was estimated from the oil press load, which was calibrated by the pressure-induced phase transitions of Bi (2.55 GPa and 2.69 GPa) and Ba (5.5 GPa). A pyrophyllite composite block (32.5 mm × 32.5 mm × 32.5 mm) was used as the pressure transfer medium. A mixture of MgO and ZrO_2_ was used as the thermal insulating tube. The B_4_C–rGO composites were sintered at 1200–1600 °C under 5.0 GPa for 10 min.

The phase compositions were examined by X-ray diffraction (XRD) with a Cu-Kα X-ray beam (λ = 1.5418 Å). The microstructure and crack path were characterized by scanning electron microscopy (SEM, FESEM JEOL-6700F, JEOL Japan Electronics Co., Ltd., Tokyo, Japan) and high-resolution transmission electron microscopy (HRTEM, JEM-2200FS, JEOL Japan Electronics Co., Ltd., Tokyo, Japan). Raman spectrums were collected on the Renishaw inVia Raman system with an excitation laser wavelength of 514.5 nm. The relative densities were measured by the Archimedes method [24]. Vickers hardness measurements were carried out on a micro-indentation hardness equipment (HV-1000ZDT, Shanghai Juhui Instrument Manufacturing, Co., Ltd., Shanghai, China) with different load forces (0.98 N~9.8 N). The applied load (P) and HV were determined by the following equation:

HV = 1854.4*P*/*d*^2^ (1)

where d is the mean of the two diagonals of the indentation. The dwelling time under the peak load was 15 s, and each sample was tested five times. The fracture toughness (K_IC_) was estimated by indentation cracking, according to the Anstis formula [25]:

K_IC_ = 0.016(E/HV)^1/2^(P/C^3/2^) (2)

where E is the Young’s modulus of the composite (GPa), HV is the Vickers hardness of the composite (GPa), P is the applied load (N), and C represents the crack length from the indented center to the tip of the emanated cracks (mm). The elastic constants of two layers of the graphene under different pressures were calculated by a CASTEP simulation. The Perdew Burke–Ernzerhof (PBE) function in generalized gradient approximation (GGA) and ultrasoft pseudopotentials were used as the exchange–interconnection energy. The first Brillouin region (K point) of the graphene was divided into a 7 × 7 × 2 Monkhorst–Pack grid for batch calculation. The plane wave truncation energy was set to 400 eV, the self-consistent accuracy was set to 2 × 10^−6^ eV, and the residual force acting on each atom did not exceed 0.006 eV/Å.

## 3. Results and Discussion

**Figure 1:**
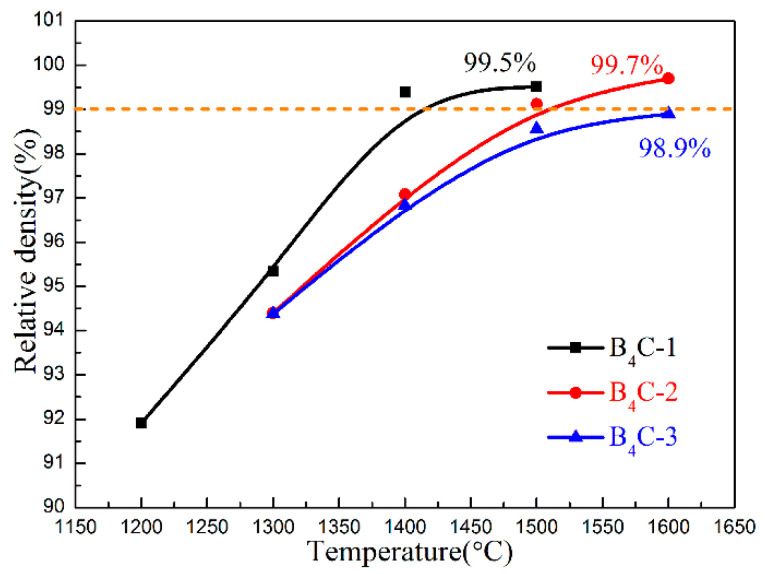
Relative density of the B_4_C–rGO composites with different rGO contents synthesized at different temperatures.

To enhance the interface strength and friction between B_4_C and rGO, a synthesis of B_4_C–rGO composites was carried out under a pressure of 5 GPa using the HPHT method. High pressure facilitates the formation of a strong bond between B_4_C and rGO. Figure 1 presents the relative densities of samples with different rGO contents at different sintering temperatures. For the B_4_C-1 *vol%* rGO sample, the relative density was only 92% at a sintering temperature of 1200 °C. When the sintering temperature was raised to 1400 °C, the relative density reached above 99%, and it remained relatively constant as the sintering temperature increased to 1500 °C. The high density was attributed to the fact that high pressure can reduce grain spacing, can eliminate pores, and can promote full contact between grains [26]. Moreover, rGO possesses self-lubricating properties that promote the deformation and sliding of B_4_C grains, accelerating the densification process [27]. For the samples with 2 *vol%* rGO and 3 *vol%* rGO, higher sintering temperatures also led to higher relative densities. The B_4_C-2 *vol%* rGO sample required a higher temperature of 1500 °C to achieve a high relative density above 99%, while the maximum value of relative density for the 3 *vol%* rGO sample was only 98.9%, even at a temperature of 1600 °C. This indicates that high temperatures can promote stronger interactions and better bonding between B_4_C and rGO. However, the relative density decreased with an increase in the rGO content, which was primarily due to the introduction of more pores created by more rGO content.

**Figure 2:**
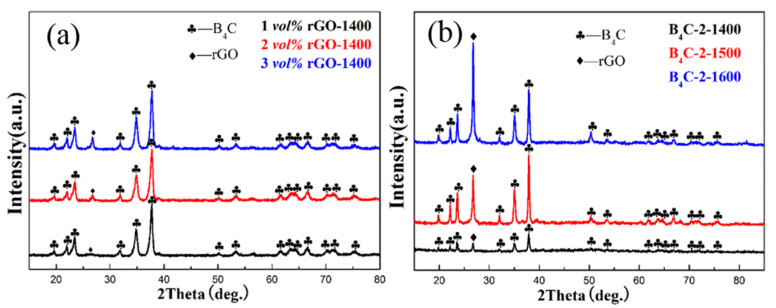
XRD patterns of as-sintered B_4_C–rGO composites. (**a**) Samples with different rGO contents synthesized at 5 GPa/1400 °C/10 min; (**b**) B_4_C-2 *vol%* rGO composites synthesized at 1400–1600 °C.

To characterize the structure of B_4_C and rGO after HPHT sintering, XRD was performed on samples with different sintering temperatures and rGO contents. Figure 2 displays the XRD patterns of samples with different rGO contents and sintering temperatures. It can be observed that the main peak of B_4_C (PDF no. 35-0798) appears in the XRD pattern, and no peaks corresponding to the initial GO were detected after HPHT sintering. However, diffraction peaks corresponding to rGO were observed near 2θ = 26°, indicating that the GO had been completely reduced to rGO after HPHT sintering.

**Figure 3:**
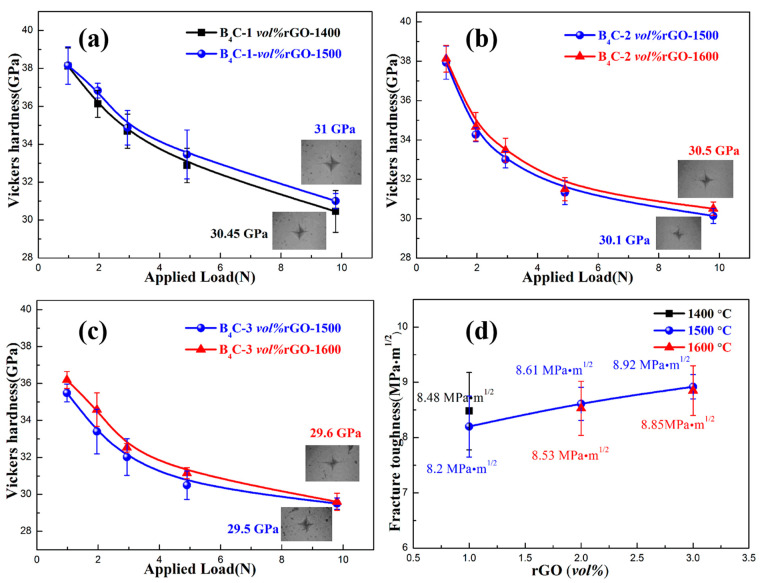
Hardness and fracture toughness profiles of B_4_C–rGO composites. (**a**–**c**) Influence of loading force and temperature on the Vickers hardness for samples with different rGO contents; the insets in (**a**–**c**) are optical microscopic images of the Vickers indentation at a 9.8 N load; (**d**) fracture toughness versus rGO content and temperature.

Revealing the hardness and toughness of B_4_C–rGO composites is crucial for understanding their intrinsic mechanical properties. Vickers hardness and indentation toughness tests were conducted on all synthesized samples. However, the hardness values were unreliable due to indentation fractures in samples with a relative density below 98%. Thus, only the hardness and toughness values for samples with a relative density above 98% are displayed in Figure 3. It can be observed that the hardness convergence value of the B_4_C-1–1500 composite is 31 GPa under a 9.8 N load. As the rGO content increases from 1 *vol%* to 3 *vol%*, the hardness decreases slightly from 31 GPa to 29.5 GPa. The reduction in hardness was mainly attributed to the lower relative density of the high-rGO-content sample. Nevertheless, the B_4_C-2-1500 composite still maintains a high hardness value of 30.1 GPa. With an increase in rGO content, the toughness of the composite increases gradually. For the sample with 2 *vol%* of rGO content, the fracture toughness reaches a maximum value of 8.6 MPa·m^1/2^, which is four times higher than that of pure B_4_C (2.2 MPa·m^1/2^) [26].

**Figure 4:**
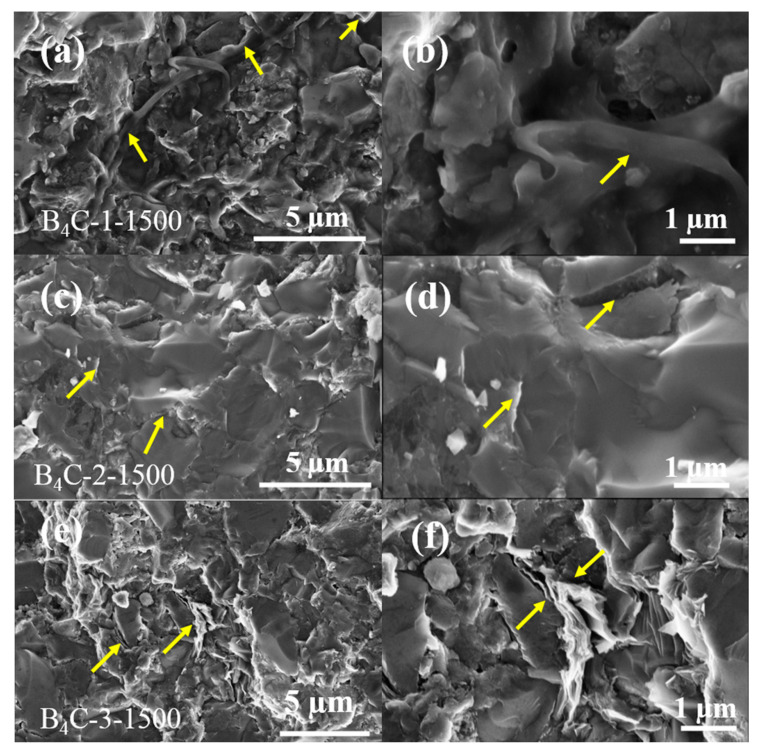
SEM images of fracture surface for samples with different rGO contents ((**a**) 1 *vol%*, (**c**) 2 *vol%*, and (**e**) 3 *vol%*) synthesized at 5 GPa/1500 °C/10 min; (**b**,**d**,**f**) shown at a higher magnification.

To explore the toughening mechanism of rGO, the fracture surface of samples with different rGO contents synthesized at 5 GPa/1500 °C/10 min was characterized by SEM. It can be seen that the curved and plate-shaped rGO distributed in the composite, as shown in Figure 4a,c,e marked by yellow arrows. With an increase in rGO content, more multiple layers of curved rGO were detected at the grain boundaries of B_4_C, as shown in Figure 4f. The folds of rGO can play a significant role in enhancing the mechanical interlocking and stress transferring of the matrix [28,29,30]. In contrast to the grain boundaries in pure-phase B_4_C ceramics, the embedded rGO interface slip is more easily activated due to the multi-layered nature of rGO. Therefore, rGO with a large specific area interface can form a lubricant network to resist external stress. This is similar to the toughening effect of amorphous carbon layers in nanocrystalline B_4_C, where rGO can act as a lubricant to promote grain boundary slips and enhance the fracture toughness of brittle B_4_C ceramics [31]. Therefore, the pull-out of rGO consumes more energy than conventional reinforcements, which effectively enhances its resistance to crack propagation.

**Figure 5:**
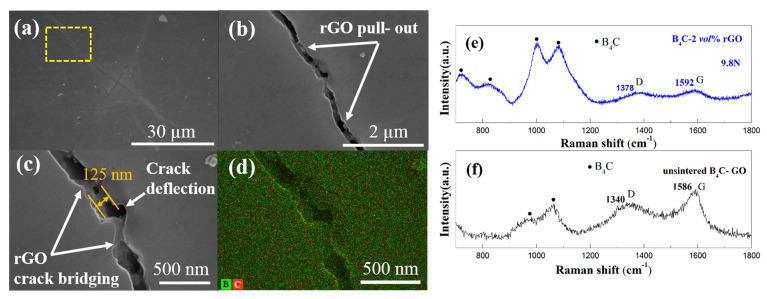
(**a**) SEM image of Vickers hardness indentation produced at a load of 9.8 N on the polished surface of B_4_C-2 *vol%* rGO sample synthesized at 5 GPa/1500 °C/10 min; (**b**) pull-out of rGO; (**c**) crack bridging and deflection; (**d**) EDS hierarchical image of element distribution in (**c**); (**e**) Raman spectra of B_4_C-2 *vol%* rGO sample, taken from a crack caused by Vickers indentation; (**f**) Raman spectra of un-sintered mixed powers.

To further analyze the toughening mechanism of rGO, the fracture pathways of the Vickers indentation cracks were studied. Figure 5a shows a polished-surface Vickers indentation SEM of the B_4_C-2 *vol%* rGO sample under a 9.8 N load. Unlike the long and straight crack propagation path in pure B_4_C, the Vickers indentation crack of the B_4_C-2 *vol%* rGO composite exhibits a tortuous crack propagation path. Several energy dissipation mechanisms, including crack deflection, rGO pull-out, and crack bridging, can be clearly seen, as shown in Figure 5b–d. According to the Raman characterization shown in Figure 5e,f, the typical Raman G peak and D peak of graphene still existed after HPHT, indicating that rGO is stable and can play a toughening role. These toughening mechanisms can consume more crack energy, leading to an enhancement in toughness. However, the bridging and pull-out of the two-dimensional rGO is distinctive from other one-dimensional carbon nanotubes and fibers. Its unique advantages of a high aspect ratio, large specific surface area, and folded and curved surfaces enable rGO’s increase in interface friction effectively and achieve the best bridging and pull-out toughening effects. Furthermore, the internal defects in rGO can enhance chemical bonding with the B_4_C matrix [32]. Additionally, the curved and folded rGO at grain boundaries is more likely to wrap around the B_4_C matrix grains, which is beneficial for generating additional mechanical interlocking and makes it difficult to separate from the B_4_C matrix, thereby enhancing the ability of stress transfer [15].

**Figure 6:**
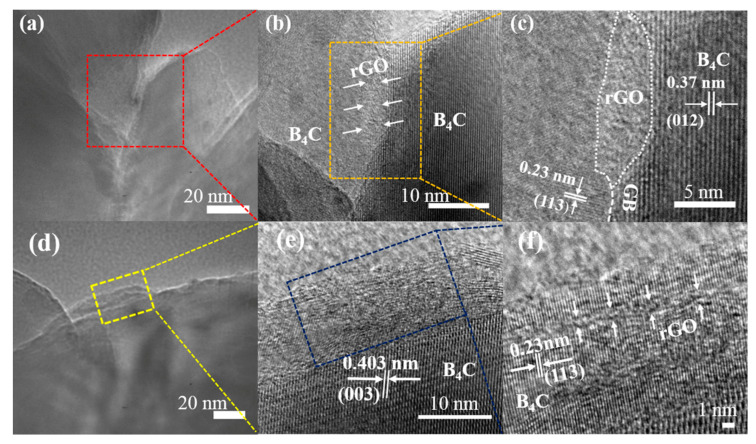
(**a**,**d**) TEM images of B_4_C-*2 vol%* rGO composite synthesized at 5 GPa/1500 °C/10 min; (**b**,**e**) HRTEM image of the square area in (**a**,**d**), respectively; (**c**,**f**) higher magnification of (**b**,**e**).

The interface is an important transition zone between rGO and the B_4_C matrix, and an appropriate interface strength is also the basis for the formation of crack bridging and other toughening mechanisms. In order to further explore the toughening mechanisms of rGO, TEM tests were performed to study interface bonding. As shown in Figure 6a–c, the folded and curved rGO appeared between the (012) and (113) crystal planes of B_4_C. When the rGO is at the grain boundaries of the B_4_C matrix, these matrix particles adjacent to the rGO can exert pressure on it, causing the rGO to bend and embed into the matrix particles. Thus, the bonding between rGO and the B_4_C matrix particles can be strengthened. Therefore, more crack energy dissipation is achieved by increasing the contact area and the friction between the rGO and the B_4_C matrix during the pull-out process [33]. Additionally, folded and curved rGO is found to be embedded into the B_4_C matrix particles, as shown in Figure 6d–f. Depending on the large specific surface area and due to the excellent surface roughness of rGO, the interface strength between rGO and B_4_C can be further enhanced by embedding the matrix particles. Thus, the pull-out of folded and layered rGO can consume more crack energy.

**Figure 7:**
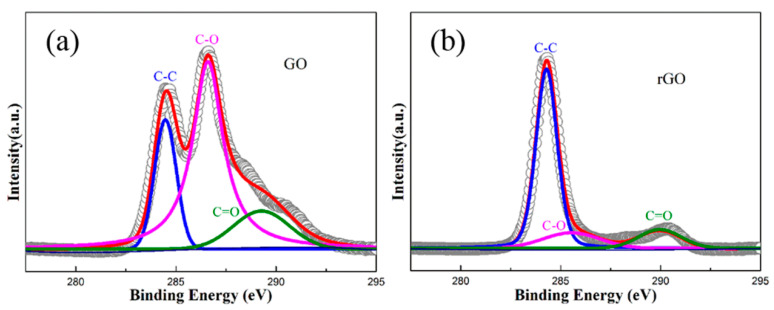
C 1s XPS spectra of GO powders (**a**) and rGO after HPHT sintering at 5 GPa/1500 °C/10 min (**b**).

The structural stability of GO after sintering is crucial for toughening, and an XPS analysis enables us to further examine the reduction process of GO. Figure 7 shows the XPS spectra of the initial GO and the rGO after HPHT sintering. It can be observed that the un-sintered GO exhibits three overlapping peaks at 283.5 eV, 285.6 eV, and 287.3 eV, corresponding to the C–C, C–O, and C=O bonds, respectively [11]. After HPHT sintering, the C 1s spectrum of GO shifts from a double peak to a single peak, with an increase in the intensity of the C–C single bond peak. Additionally, a significant reduction in the C–O single bond peak is found, while the C=O double bond peak undergoes a shift towards the higher frequency direction. The obvious changes in the chemical bonds in the XPS spectra were attributed to the presence of numerous oxygen functional groups in GO [11]. When the temperature reaches the activation energy required for the cleavage of the carbon–oxygen bonds in the oxygen functional groups during the HPHT sintering process, the chemical bonds are disrupted and broken, leading to the evaporation of oxygen functional groups on the surface of the GO. This indicates restoration of the sp2 bonds in rGO, which suggests that the GO completely transformed into rGO after HPHT sintering [32].

**Figure 8:**
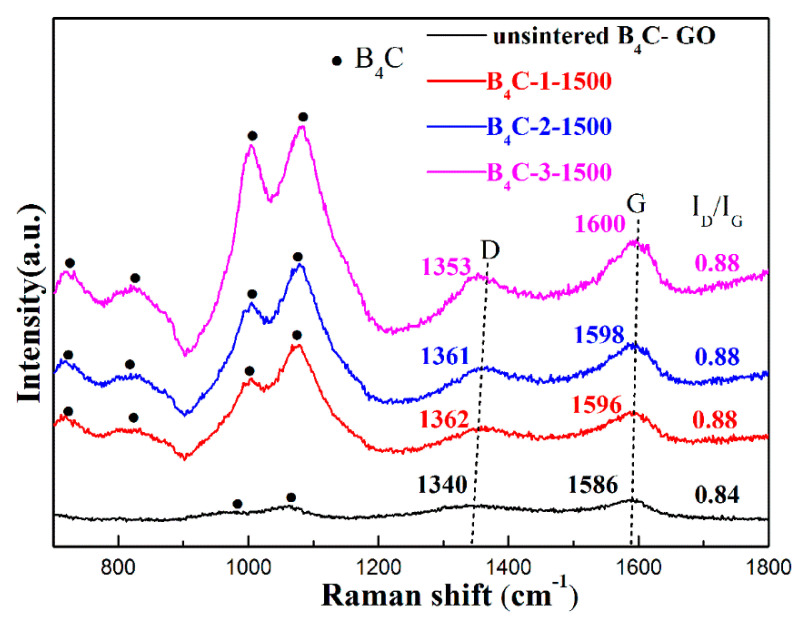
Raman spectra of un-sintered mixed powers and B_4_C–rGO composites with different rGO contents synthesized at 5 GPa/1500 °C/10 min.

Raman spectroscopy is an effective technique for characterizing carbon materials, and significant structural changes from GO to rGO during the sintering process are also reflected in Raman spectra. Figure 8 shows the Raman spectra of the B_4_C–GO-mixed un-sintered powders and samples with different rGO contents after HPHT sintering. Generally, a D peak at 1344 cm^−1^ is typically associated with a lattice disorder, while a G peak at 1590 cm^−1^ corresponds with an in-plane C–C symmetric stretching vibration in graphene sheets [34]. It is evident that the D and G peaks of the samples after HPHT sintering are enhanced compared to the un-sintered mixed powders. The increase in the intensity of the D and G peaks may be attributed to the presence of more interfacial graphene with an increase in GO in the composite, indicating that rGO remains in the B_4_C matrix after HPHT sintering. Furthermore, no broadening D and G peaks appeared after HPHT sintering, suggesting that rGO maintains good structural stability. The intensity ratio of the D peak to the G peak (I_D_/I_G_) can also be used to assess the degree of sp2-based carbon structure defects or disorders. It can be seen that the I_D_/I_G_ of the samples increased slightly after sintering, which could be attributed to the cleavage of the C–O–C bonds during the thermal reduction process, leading to the simultaneous reconstruction of adjacent C=C double bonds. Additionally, the G peak is observed to shift towards higher frequencies after HPHT sintering. Compared to the initial G peak in the un-sintered samples, the G peak of the B_4_C-1 *vol%* rGO composite has a shift value of ∆X_1_ = 10. According to the high-pressure Raman spectrum of graphene reported by Proctor et al. [35], the G peak shift of graphene under a 5 GPa pressure is ∆X = 21.3. Consequently, the prestress in the 1 *vol%* rGO sample can be estimated as (∆X_1_/∆X) × 5 = 2.3 GPa. When the rGO content increases to 3 *vol%*, the calculated prestress value is 3.3 GPa. It is evident that the prestress increases with an increase in rGO content, leading to an improvement in toughness, gradually. This compressive stress results in a strong interface bond between rGO and B_4_C, which effectively increases the bearing capacity and the friction force between the rGO and B_4_C matrix during the pull-out process. Therefore, the role of rGO bridging and pull-out is enhanced, forming a stress-strengthened bridging and pull-out toughening mechanism.

The mechanical properties of graphene under different pressures can be further estimated by theoretical calculations. Table 1 presents the elastic constants of two layers of graphene at different pressures (0, 1, 2, and 3 GPa). It can be seen that all elastic constants increase with an increase in pressure. Compared with no pressure, the shear modulus and Young’s modulus both increase by 21% under a 3 GPa pressure. This indicates that prestress significantly enhances the mechanical properties of hollow graphene by improving the interaction strength, leading to the high toughness of the composite. Thus, a toughening phase with a larger hollow-space structure and strong mechanical properties can store more internal stress by HPHT, which is able to further strengthen the pull-out toughening mechanism and can acquire higher toughness values.

**Figure 9:**
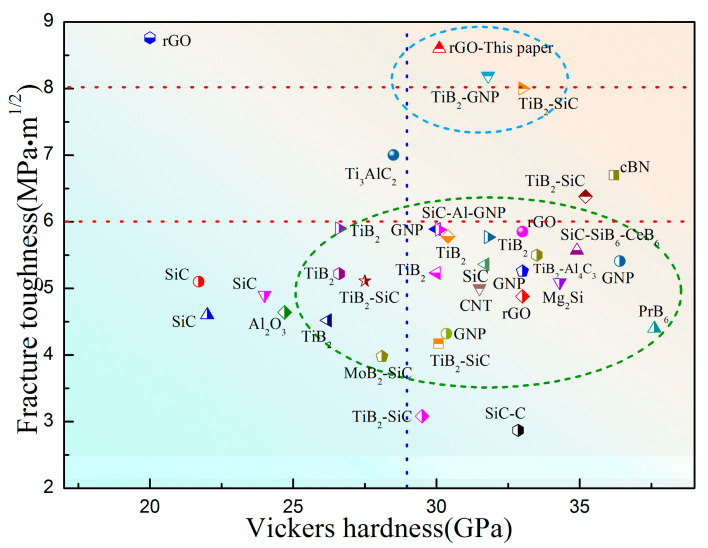
Plot of the Vickers hardness and fracture toughness of this work in comparison with previous reports.

Figure 9 shows a comparison of the hardness and toughness values of the B_4_C matrix composite synthesized in this paper and reported by other research studies [8,9,10,11,12,17,18,24,32,36,37,38,39,40,41,42,43,44,45,46,47,48,49,50,51,52,53,54,55,56,57,58,59,60,61,62]. It can be seen that most of the composites could achieve a toughness value close to 6 MPa·m^1/2^ and a high hardness value of more than 30 GPa but were rarely able to exceed 8 MPa·m^1/2^ and maintain a high hardness of 30 GPa. This study demonstrates that two-dimensional hollow graphene toughening phases can retain prestress by HPHT sintering with B_4_C. This prestress further enhances the interfacial strength between the rGO and B_4_C matrix, forming a strengthened bridge and pull-out toughening mechanism. These findings provide new insights for the design of high-toughness B_4_C–graphene composites.

## 4. Conclusions

A new method to reinforce B_4_C by introducing prestress in B_4_C–rGO composites was realized by HPHT. rGO maintains the layered structure of graphene after HPHT sintering. The B_4_C-2 *vol%* rGO composite achieved optimal hardness and toughness values of 30.1 GPa and 8.6 MPa·m^1/2^, respectively, which was almost 4 times higher than pure B_4_C. Crack bridging, crack deflection, and pulling out induced by rGO are the main toughening mechanisms. HPHT sintering results in internal stress between B_4_C and rGO, and this internal stress increases from 2.3 GPa to 3.3 GPa with an increase in rGO content from 1 *vol%* to 3 *vol%*. This high internal stress effectively enhances the interfacial strength and interface friction between the folded layers of rGO and the B_4_C matrix, leading to a significant enhancement in toughness.

## Figures and Tables

**Table 1:** Mechanical properties of two layers of graphene under different pressures.

Elastic Constant (GPa)	0 GPa	1 GPa	2 GPa	3 GPa
Bulk modulus	224.1	226.7	269.7	271.3
Shear modulus	180.3	204.3	214.6	218.1
Young’s modulus	426.7	484.0	509.0	516.1

## Data Availability

The original contributions presented in the study are included in the article, further inquiries can be directed to the corresponding authors.

## Supplementary Materials

The following supporting information can be downloaded at: https://www.mdpi.com/article/10.3390/ma17235838/s1, Figure S1: Initial mixed powders with different GO content.
