# Supplementary material for: Reduced Graphene Oxide Reinforces Boron Carbide with High-Pressure and High-Temperature Sintering
Source: Materials (Basel). 2024 Nov 28;17(23):5838. doi: 10.3390/ma17235838 (PMC11643588; doi:10.3390/ma17235838)
Supplement: Supplementary file 1 [file materials-17-05838-s001.zip › materials-3312513-supplementary.pdf]

Reduced Graphene Oxide Reinforced Boron Carbide by High Pressure and High Temperature Sintering

Xiaonan Wang [1,2], Dian Zhen Wang [2], Kaixuan Rong [2], Qiang Tao [2,*] and Pinwen Zhu [2,*]

Affiliations

1. Key Laboratory of Functional Materials Physics and Chemistry of the Ministry of Education, Jilin Normal University, Changchun, 130103, China
2. Synergetic Extreme Condition High-Pressure Science Center, State Key Laboratory of Superhard Materials, College of Physics, Jilin University, Qianjin Street, Changchun, 130012, People's Republic of China

*Correspondence: Email: zhupw@jlu.edu.cn and qiangtao@jlu.edu.cn

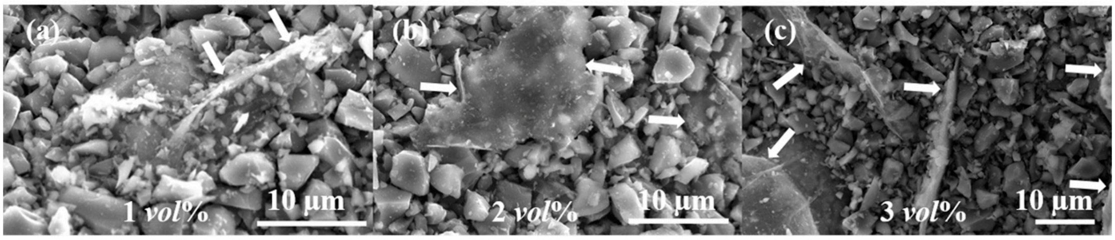

Figure S1. Initial mixed powders with different GO content.
